# First record of the fungal genus *Neodevriesia* Quaedvl. & Crous (Ascomycota, Dothideomycetes, Neodevriesiaceae) isolated from scleractinian corals of Perhentian Islands, Malaysia

**DOI:** 10.3897/BDJ.10.e81533

**Published:** 2022-05-18

**Authors:** Li Chuen Lee, Mohammed Rizman-Idid, Siti Aisyah Alias, Kishneth Palaniveloo, Haifeng Gu

**Affiliations:** 1 Institute of Ocean and Earth Sciences, Institute for Advanced Studies Building, Universiti Malaya, Kuala Lumpur, Malaysia Institute of Ocean and Earth Sciences, Institute for Advanced Studies Building, Universiti Malaya Kuala Lumpur Malaysia; 2 Third Institute of Oceanography, Ministry of Natural Resources, Xiamen, China Third Institute of Oceanography, Ministry of Natural Resources Xiamen China

**Keywords:** marine fungi, hard corals, Dothideomycetes, DNA sequences, Perhentian Islands

## Abstract

Fungal species members of the genus *Neodevriesia* have been known to occur in marine environments. This report documents the first record of the fungal genus *Neodevriesia* isolated from scleractinian corals. Three isolated strains were identified from a phylogenetic tree that was constructed, based on the nuclear ribosomal internal transcribed spacer and partial large subunit (ITS + LSU) DNA sequences. Isolates were closely related to both *Neodevriesiashakazului* (Crous) Crous and *Neodevriesiaqueenslandica* (Crous, R.G. Shivas & McTaggart) Crous, but formed a distinct clade with strong support that implies a potentially genetic variant of a known species or even a novel species. These findings contribute to the fungal diversity checklist in Malaysia and knowledge about marine fungi associated with scleractinian corals.

## Introduction

The fungal genus *Neodevriesia* Quadvl. & Crous 2014 (family Neodevriesiaceae) was established, based on the type *Neodevriesiahilliana* (Crous & U. Braun) Quaedvl. & Crous to accommodate *Devriesia*-like species within the clade *Devriesia* sensu lato (s. lat.) (Crous et al. 2009, Frank et al. 2010, Quaedvlieg et al. 2014). *Neodevriesia* is described having morphological characters of medium brown and unbranched conidiophores, thick-walled, medium brown, rarely septate conidia, short and mostly unbranched conidial chains and the absence of chlamydospores (Quaedvlieg et al. 2014).

More than 20 species from the genus *Neodevriesia* have been described from a broad range of habitats across wide geographic regions. For example, the extremophilic fungi *Neodevriesiabulbillosa* Egidi & Zucconi was isolated from limestone in the Malloracan mountain range (Ruibal et al. 2005). The mycoparasitic *Neodevriesiacoryneliae* Crous & A.R. Wood was found growing on the ascomata of *Coryneliauberata* Fr., which is a common pathogenic fungi of *Podocarpus* spp., known commercially as podo or East African yellow-wood (Crous et al. 2014). Despite being first and primarily described as a terrestrial species, subsequent discoveries have since expanded the distribution of *Neodevriesia* to include the marine environment. Wang et al. (2018) have isolated and described several fungal species from marine algae in China; *Neodevriesiacladophorae* M.M. Wang & W. Li from *Cladophora* sp. (Chlorophyta) and *Ahnfeltiopsis* sp. (Rhodophyta); and *Neodevriesiagrateloupiae* M.M. Wang & W. Li from *Grateloupia* sp. (Rhodophyta) and *Blidingia* sp. (Chlorophyta). *Neodevriesiaaestuarina* M. Gonçalves & A. Alves was isolated from an estuary in Ria de Aveiro, Portugal (Crous et al. 2020). In an effort to examine marine fungi, we investigated fungi associated with scleractinian corals across coral reefs surrounding the Perhentian Islands, Malaysia. Herein, we report the first record of the fungal genus *Neodevriesia* isolated from several scleractinian corals of Malaysia, based on preliminary identification of their morphology and DNA sequences.

## Materials and Methods

**Figure 1.**
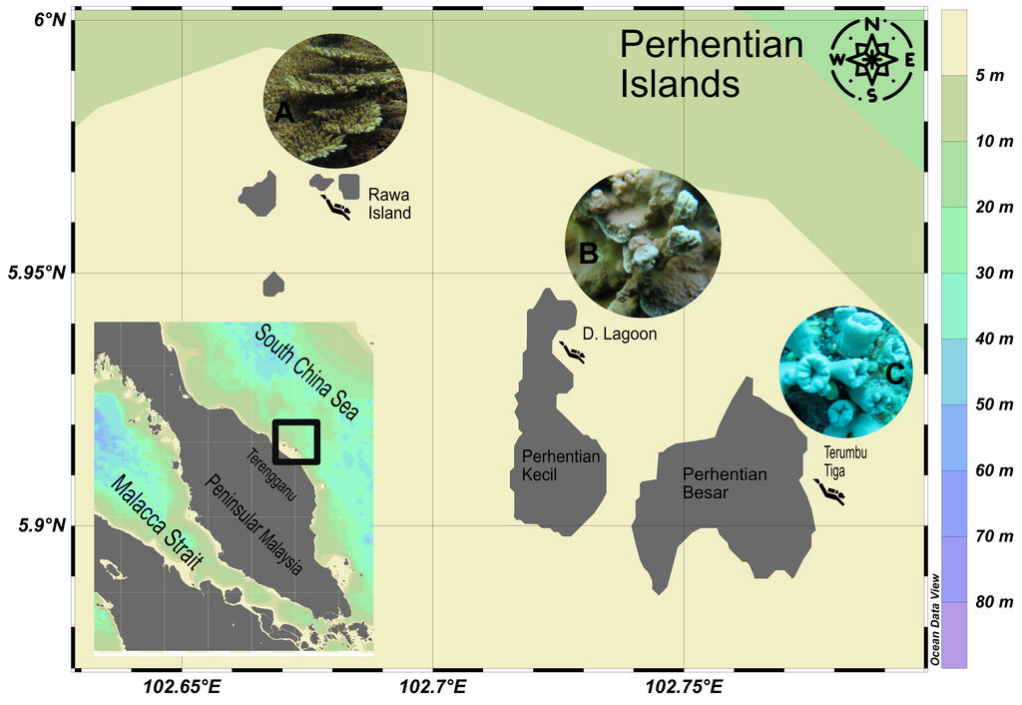
Locations of the three sampling sites (Rawa Island, D’Lagoon and Terumbu Tiga) in the Perhentian Islands, Terengganu, Peninsular Malaysia where scleractinian coral samples A) *Acropora* sp., B) *Porites* sp. and C) *Tubastraea* sp., were obtained by SCUBA diving.

Samples of scleractinian coral colonies comprising *Acropora* sp., *Porites* sp., *Tubastraea* sp. were collected on 14 May 2017, from the coral reefs of Perhentian Islands, located at the east coast of Terengganu, Peninsular Malaysia. Sampling sites were Rawa Island (5°57'38.28"N, 102°40'54.59"E), D'Lagoon of Perhentian Besar (5°55'56.34"N, 102°43'23.21"E) and Terumbu Tiga of Perhentian Kecil (5°54'2.76"N, 102°46'25.69"E) (Fig. 1). Scleractinian coral samples were collected by SCUBA diving and initially identified underwater using the Coral Finder Guidebook 3.0 (Kelley and Pears 2016). The scleractinian corals were photographed underwater using an Olympus TG-5 digital camera for subsequent identification, based on Coral of the World (Vol. 1-3) (Veron and Stafford-Smith 2000). Scleractinian coral fragments (4 ~ 6 cm) were cut and placed into separately labelled plastic bags with seawater. Once out of the water, coral samples were divided into two sets of labelled 50 ml Falcon tubes; one set containing absolute ethanol for preservation and the other set in natural seawater for isolation of coral-associated fungi. The coral samples were kept cold on ice until transported back to the laboratory and stored in 8^o^C.

Isolation of coral-associated fungi was conducted following modifed methods of Li et al. (2014). Coral fragments were dipped in 90% ethanol for 3 mins for surface sterilisation and rinsed with sterilised artificial seawater. The coral fragments were ground and mixed with sterilised artificial seawater. The slurry was transferred into a Falcon tube and vortexed for 5 minutes. Ten-fold serial dilutions (n = 3) were made from the slurry. One hundred μl of each dilution was spread on to Petri dishes containing Corn Meal Agar (CMA) prepared with 70% seawater containing Streptomycin (n = 3). The plates were incubated at 26^o^C for 5 days until the manifestation of mycelium. The fungal strains were then isolated and sub-cultured on Czapek-Dox Agar (CDA). CDA was used for its sodium nitrate as the sole source of nitrogen that promotes growth. The pure fungal isolates were kept at 8^o^C for long-term storage.

Pure fungal isolates were identified, based on the morphology of the spores and hyphae. Pure isolates were cultured under continuous normal light on CDA for three weeks at 26°C. Mycelial plugs (5 mm diameter) were cut from colony margins and placed in 9-cm-diameter Petri dishes (n = 3). CDA plugs (1 cm × 1 cm) were placed in clean Petri dishes, each agar plug was embedded with conidia and a coverslip was placed over each plug according to Riddell (1950). After 7 days, the micrographs were taken under a Nikon Eclipse Light microscope.

Genomic DNA were isolated from strains that were incubated at 26^o^C in Potato Dextrose Broth (PDB) for 7 days. Mycelia were harvested by filtering the broth and genomic DNA was extracted using BioTeke Plant DNA purification kit (BioTeke, China) following the manufacturer’s protocol. Two loci were amplified; the nuclear ribosomal internal transcribed spacer (ITS) using the primer pair ITS 5 (forward): 5’-GGA AGT AAA AGT CGT AAC AAG G-3’) and ITS 4 (reverse): 5’-TCC TCC GCT TAT TGA TAT GC-3’ (White et al. 1990); and the partial large 28S gene (LSU) including the D1-D2 domains using primer pairs NL1 (forward): 5’-GCA TATCAA TAA GCG GAG GAA AAG-3’ with NL4 (reverse): 5’-GGT CCG TGT TTC AAG ACGG-3’ (O’Donnell 1993). PCR amplification was performed in 20 μl total volume with a final concentration of 1 X Reaction Buffer (GeNet Bio) (2 mM Tris-HCl, pH 9.0, 2 mM MgCl_2_), 0.25 mM dNTPs mixture, 2.5 U Taq DNA Polymerase (GeNet Bio), 0.1 mM of each primer and 10 ng of gDNA template. PCR thermal cycling was performed using Applied Biosystems™ SimpliAmp™ Thermal Cycler following these profile conditions; ITS : 95^o^C for 5 mins, followed by 30 cycles of 95^o^C for 1 min, 55^o^C for 1 min, 72^o^C for 1.5 mins with a final extension step of 72^o^C for 10 mins (Kwiatkowski et al. 2012); LSU : 94^o^C for 2 mins, followed by 30 cycles of 94^o^C for 15 secs, 55^o^C for 30 secs, 68^o^C for 2 mins with a final extension step of 68^o^C for 5 mins (Kwiatkowski et al. 2012). Amplicons were sequenced by First Base Sequencing Services (Malaysia) using BigDye® Terminator v.3.1 cycle (Applied Biosystem, USA).

DNA sequence reads and chromatograms of the fungal isolates were inspected using Sequence Scanner v.1.0 (Applied Biosystem), edited using BioEdit ver. 7.2 (Hall 1999) and aligned with Clustal-X 2.0 (Thompson et al. 1994, Larkin et al. 2007) to assemble the contiguous sequence for each fungal isolate. BLASTn (Altschul et al. 1990) was used to search for both homologous ITS and LSU sequences in GenBank. Fifty-nine reference sequences (Crous et al. 2018) (Suppl. material 1) were obtained from GenBank for phylogenetic analysis. Multiple sequence alignment was conducted with Multiple Alignment using Fast Fourier Transform (MAFFT) (Katoh et al. 2017) with L-INS-i algorithms and default parameters. The aligned sequences were trimmed and analysed for their genetic distances using Molecular Evolutionary Genetic Analysis (MEGA X) (Kumar et al. 2018). Maximum Likelihood (ML) and Bayesian Inference (BI) were used for phylogenetic inferences using RAxML-NG (Kozlov et al. 2019) and MrBayes v.3.2.7 (Ronquist et al. 2012), respectively, on an online platform Cyberinfrastructure for Phylogenetic Research (CIPRES) Science Gateway V. 3.3 (Miller et al. 2010). The datasets of ITS and LSU were concatenated (ITS + LSU) for the multi-locus phylogenetic analyses using SequenceMatrix (Vaidya et al. 2011).

The best nucleotide substitution model for the concatenated sequences was GTR+I+G4 according to the Akaike Information Criterion (AIC) from the Modeltest-NG (Darriba et al. 2019) and were subsequently incorporated into ML and BI analysis. ML was conducted using 1,000 bootstrap replicates with bootstrap values (BS) calculated. For the BI analysis, Markov Chain Monte Carlo (MCMC) was used to estimate the posterior probability (PP) distribution. Iterations were performed at 10,000,000 generations per run, sampling frequency of 1,000 and PP was estimated with 25% burn-in and bootstrap support was calculated, based on 1,000 iterations.

## Results and Discussion

**Figure 2.**
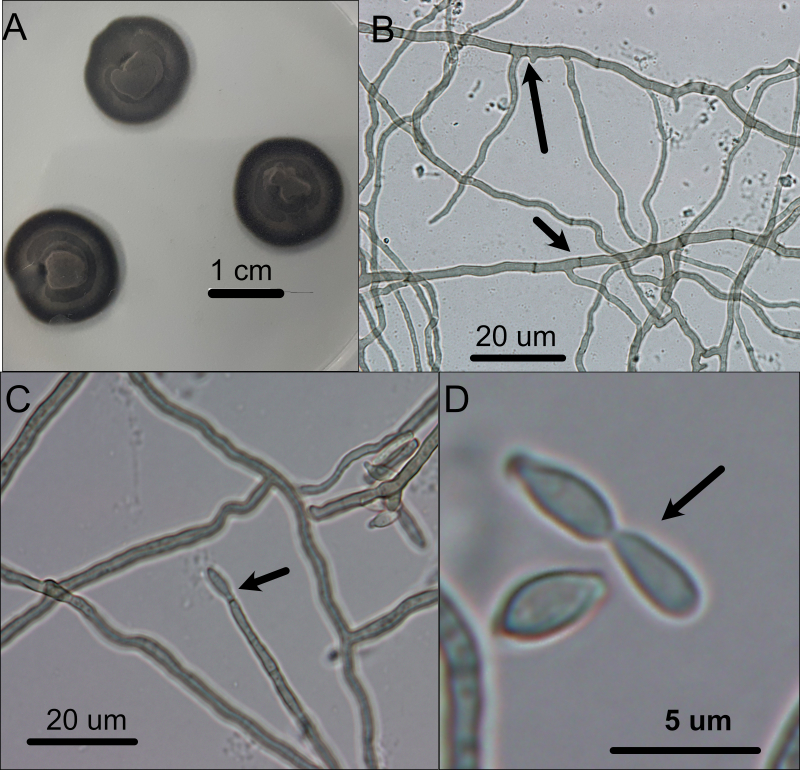
Light micrographs of colonies and fungal morphologies of *Neodevriesia* strain PERF1811. **A** Fungal colonies appearance cultured in CDA; **B** Septate hyphae (arrowed); **C** Conidiophore (arrowed) with conidia at the tip; **D** Conidia in a simple chain (arrowed).

Three fungal strains, PERF0613 (from *Acropora* sp.), PERF1511 (from *Porites* sp.) and PERF1811 (from *Tubastraea* sp.) were isolated and morphologically identified as *Neodevriesia* spp. These colonies were erumpent, spreading, with moderate aerial mycelium and smooth, lobate margin, reaching 16 mm diameter after 1 week at 26°C. Mycelium was pale olivaceous-grey in the centre, iron-grey in the outer region and iron-grey in the reverse. Mycelium consisted of brown, smooth, branched, septate, ~ 1.5 μm diameter hyphae. Conidiophores were erected, mononematous, subcylindrical, unbranched. Conidia in simple chains, hyaline, smooth, subcylindrical to narrowly fusoid, tapering at ends to truncate hila, 0.5–1 μm diameter (Fig. 2).

**Figure 3.**
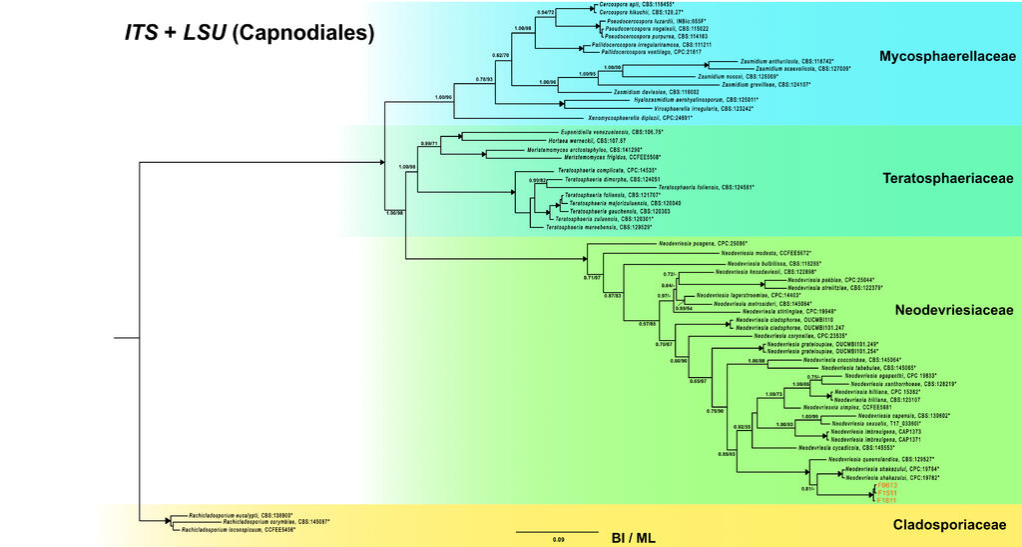
Consensus tree (BI, ML) based on BI topology of *Neodevriesia* with closely related genera using concatenated ITS and LSU sequences. Only clades with PP > 0.50 and BS > 50% are indicated at the nodes. ‘Triangle’ at the node indicated support of 1.00/100. Tree was rooted with *Rachlcladosporium* spp. from the family Cladosporiaceae. Families are indicated with coloured blocks to the right of the tree. Isolation code numbers are indicated behind the species names. ‘*’ indicated the TYPE and Ex-TYPE specimens. Fungal strains from this study are indicated in orange.

The phylogenetic tree analysis of ITS + LSU (1397 bp) confirmed that the strains PERF0613, PERF1511 and PERF1811 belong to the genus *Neodevriesia*. The strains were closely related to *Neodevriesiashakazului* and *Neodevriesiaqueenslandica* (PP = 1.00, BS = 100) (Fig. 3). Although the marine fungal strains formed a sister group to *N.shakazului*, they formed a distinct clade (PP = 1.00, BS = 100) with a long branch length (2.83% sequence divergence) (Suppl. material 2)

Such phylogenetic affinity of the marine fungal strains were unexpected, since *N.shakazului* (Crous et al. 2011) and *N.queenslandica* (Crous et al. 2012) are terrestrial species which was first isolated and described from plants *Aloe* sp. and *Scaevolataccada*, respectively. In terms of morphology, they are indistinguishable from the strains in the present study. However, the molecular data seem to suggest they may potentially be a novel species. These *Neodevriesia* strains are the first to be isolated from the marine environment, particularly from scleractinian corals.

Despite the fungi having been long-deemed as parasitic, studies have shown that fungi are integral members of the microbiome, which allow for hard corals to thrive in oligotrophic water (Amend et al. 2011, Bourne et al. 2016, Wegley et al. 2007). Although information is still scarce and evidence to implicate the functional role of *Neodevriesia* in coral microbiome remains inconclusive, a more general functional role of nitrogen cycling has been proposed to justify the presence of fungi in hard corals (Rädecker et al. 2015). Fungal functional genes involved in nitrogen cycling were detected in various metagenomic studies on corals (Wegley et al. 2007, Kimes et al. 2010, Zhang et al. 2015, Zhang et al. 2021)

Although the three strains are genetically distinct from the other two known species (i.e. *Neodevriesiashakazului* and *Neodevriesiaqueenslandica*), it is still premature to formally describe them as a new species due to the insufficient number of fruiting bodies obtained. Hence, what we are reporting here is preliminary and, thus, requires future work that includes some measurements of additional morphological characters for the full description. Additional genetic markers, such as RNA polymerase II gene (RPB2), actin (Act) and calmodulin (Cal), will also be used to further confirm them as a novel species. It is also worthwhile to further investigate and gain insights into the ecological roles of this group of fungi in the marine environment. Nevertheless, there is no doubt that this report documents the first record of genera Neodevriesia found from scleractinian corals. These findings will contribute to the fungal diversity checklist of Malaysia (Lee et al. 2012).

## Supplementary Material

B98235FC-1F9A-5DFF-8576-D4547C9606CA 10.3897/BDJ.10.e81533.suppl1 Supplementary material 1 Accession list of the sequences used in this study Data type GenBank accession numbers (.docx) Brief description Accession numbers include details such as host, locality, isolation numbers of the sequences used for this study. File: oo_640076.docx https://binary.pensoft.net/file/640076 Li Chuen Lee

25298A92-803D-5B31-B7A2-746093834271 10.3897/BDJ.10.e81533.suppl2 Supplementary material 2 Evolutionary divergence between sequences Data type Evolutionary divergence (.csv) Brief description Estimates of evolutionary divergence between sequences conducted using the Kimura 2-parameter model. File: oo_639791.xls https://binary.pensoft.net/file/639791 Li Chuen Lee
