# Supplementary material for: First record of the fungal genus Neodevriesia Quaedvl. & Crous (Ascomycota, Dothideomycetes, Neodevriesiaceae) isolated from scleractinian corals of Perhentian Islands, Malaysia
Source: Biodivers Data J. 2022 May 18;10:e81533. doi: 10.3897/BDJ.10.e81533 (PMC9848531; doi:10.3897/BDJ.10.e81533)
Supplement: Supplementary material 1 — Accession list of the sequences used in this study [file bdj-10-e81533-s001.docx]

**Supplementary A: Accession numbers of both marker *ITS* and *LSU* with their details (Host, isolation number and locality) of the sequences used for this study.**

| Species | Isolation number | Host/Habitat | Locality | LSU | ITS |
| --- | --- | --- | --- | --- | --- |
| *N. cycadicola* | CBS 145553 (T) | *Cycas* sp. | Italy | NG_067886 | NR_165568 |
| *N. knoxdaviesii* | CBS 122898 (T) | *Protea* sp. | South Africa | MH874778 | EU707865 |
| *N. metrosideri* | CBS:145084 (T) | *Metrosideros* sp. | New zealand | NG_066296 | NR_161141 |
| *N. sexualis* | T17_03360I (T) | *Archontophoenix cunninghamiana* | New zealand | NG_067887 | NR_165569 |
| *N. tabebuiae* | [CBS:145065](http://www.cbs.knaw.nl/collections/BioloMICS.aspx?Fields=All&ExactMatch=T&Table=CBS+strain+database&Name=CBS+145065) | *Tabebuia chrysantha* | Puerto Rico | NG_066286 | NR_161127 |
| *N. agapanthi* | CPC 19833 (ET) | *Agapanthus africanus* | South Africa | NG_042688 | NR_111766 |
| *N. bulbillosa* | CBS 118285 (ET) | Rock sample | Spain | KF310029 | AY559341 |
| *N. capensis* | CBS 130602(ET) | *Protea* sp. | South Africa | JN712569 | JN712501 |
| *N. cladophorae* | OUCMBI110 | *Cladophora* sp. | China | KU578114 | KU578112 |
|  | OUCMBI101 | *Ahnfeltiopsis* sp. | China | KU578117 | KU578115 |
| *N. coryneliae* | CPC 23534 (ET) | *Corynelia uberata* | South Africa | KJ869211 | NR_132905 |
| *N. grateloupiae* | OUCMBI101 249 (T) | *Grateloupia* sp. | China | KU578120 | KU578118 |
|  | OUCMBI141 254 | *Blidingia* sp. | China | KX237688 | KX237686 |
| *N. hilliana* | CPC 15382 (ET) | *Macrozamia communis* | New Zealand | GU214414 | GU214633 |
|  | CBS 123187 | *Macrozamia communis* | New Zealand | MH874801 | NR_145098 |
| *N. imbrexigena* | CAP1371 | Decorative wall tile | Portugal | JX915749 | JX915745 |
|  | CAP1373 | Decorative wall tile | Portugal | JX915750 | JX915746 |
| *N. lagerstroemiae* | CPC 14403 (ET) | *Largerstore indiea* | USA | GU214415 | GU214634 |
| *N. modesta* | CCFEE5672(ET) | Rock sample | Italy | KF3100 26 | KF309984 |
| *N. pakbiae* | CPC25044(ET) | Unidentified fern | Thailand | KR476775 | NR_137997 |
| *N. poagena* | CPC25086(ET) | *Poa* sp. | Netherland | KR611903 | KR611885 |
| *N. queenslandica* | CBS129527(ET) | *Scaevola taccada* | Australia | KF901839 | JF951148 |

**Supplementary A: Continued.**

| Species | Isolation number | Host/Habitat | Locality | LSU | ITS |
| --- | --- | --- | --- | --- | --- |
| *N. shakazului* | CPC 19784 (ET) | *Aloe* sp. | South Africa | KC005797 | KC005776 |
|  | CPC 19782 (T) | *Aloe* sp. | South Africa | NG_042753 | NR_111825 |
| *N. coccolobae* | CBS:145064 (T) | *Coccoloba uvifera* | Puerto Rico | NG_066285 | NR_161126 |
| *N. simplex* | CCFEE 5681 (ET) | Rock sample | Italy | KF310027 | KF309985 |
| *N. stirlingiae* | CPC 19948(ET) | *Stirlingia latifolia* | Australia | KC005799 | NR_120228 |
| *N. strelitziae* | CBS 122379 (ET) | *Stelitzia nicolai* | South Africa | GU301810 | EU436763 |
| *N. xanthorrhoeae* | CBS 128219 (ET) | *Xanthorrhoea australia* | Australia | HQ599606 | HQ599605 |
| *Teratosphaeria complicata* | CPC14535 (ET) | *Eucalyptus miniata* | Australia | GQ852714 | GQ852790 |
| *T. foliensis* | CBS 124581 (T) | *Eucalyptus globulus* | Australia | KF442557 | KF442517 |
|  | CBS 121707 (E-T) | *Protea* sp. | South Africa | KF902075 | KF901728 |
| *T. mareebensis* | CBS 129529 (ET) | *Eucalyptus alba* | Australia | KF901906 | NR_156577 |
| *T. gauchensis* | CBS:120303, CMW:17331 | *Eucalyptus grandis* | Uruguay | EU019290 | EU019290 |
| *T. zuluensis* | CBS 120301 (ET) | - | South Africa | MH874640 | MH863081 |
| *T. dimorpha* | [CBS:124051, CPC 14132](http://www.cbs.knaw.nl/collections/BioloMICS.aspx?Fields=All&ExactMatch=T&Table=CBS+strain+database&Name=CBS+124051) | *Eucalyptus caesia* | New South Wales, Australia | FJ493215 | KF901575 |
| *T. majorizuluensis* | CBS 120040 | *Eucalyptus botryoides* | Australia | KF442550 | KF901581 |
| *Hortaea werneckii* | CBS 107.67 | *Homo sapien* | Portugal | EU019270 | NR_145338 |
| *Eupenidiella venezuelensis* | CBS106.75 (T) | *Eucalyptus* sp. | Venezuela | NG_059224 | NR_153971 |
| *Meristemomyces  frigidus* | CCFEE:5508 (T) | Rock | Andes | NG_059444 | NR_144973 |
| *Meristemomyces arctostaphylos* | CBS:141290 (T) | *Acrostaphylos patula* | Utah, USA | NG_058234 | NR_155391 |
| *Zasmidium grevilleae* | CBS:124107 (T) | *Grevillea decurrens* | Australia | NG_060790 | NR_156522 |
| *Z. daviesiae* | CBS:116002 | *Daviesia mimosoides* | Australia | KF901928 | KF901603 |
| *Z. scaevolicola* | [CBS:127009 (T)](http://www.cbs.knaw.nl/collections/BioloMICS.aspx?Fields=All&ExactMatch=T&Table=CBS+strain+database&Name=CBS+127009) | *Scaevola taccada* | Australia | NG_069974 | NR_156565 |
| *Z. anthuriicola* | [CBS:118742 (T)](http://www.cbs.knaw.nl/collections/BioloMICS.aspx?Fields=All&ExactMatch=T&Table=CBS+strain+database&Name=CBS+118742) | *Anthurium* sp. | Thailand | NG_069155 | NR_156521 |
| *Z. nocoxi* | [CBS:125009 (T)](http://www.cbs.knaw.nl/collections/BioloMICS.aspx?Fields=All&ExactMatch=T&Table=CBS+strain+database&Name=CBS+125009) | Litter | USA | NG_059439 | NR_156536 |
| *Cercospora apii* | [CBS:116455 (T)](http://www.cbs.knaw.nl/collections/BioloMICS.aspx?Fields=All&ExactMatch=T&Table=CBS+strain+database&Name=CBS+116455) | *Apium graveolens* | Germany | NG_069525 | NR_119525 |

**Supplementary A: Continued.**

| Species | Isolation number | Host/Habitat | Locality | LSU | ITS |
| --- | --- | --- | --- | --- | --- |
| *Cercospora kikuchii* | CBS 128.27, CPC 5068 (T) | *Glycine soja* | Japan | NG_069614 | NR_119616 |
| *Pallidocercospora irregulariramosa* | [CBS:111211](http://www.cbs.knaw.nl/collections/BioloMICS.aspx?Fields=All&ExactMatch=T&Table=CBS+strain+database&Name=CBS+111211) | *Eucalyptus saligna* | South Africa | GU214441 | KF901706 |
| *Pallidocercospora ventilago* | CPC 21817 (T) | *Ventilago denticulata* | Thailand | NG_058047 | NR_137119 |
| *Pseudocercospora luzardii* | INBio:655F (T) | *Crescentia alata* | Costa Rica | KT290167 | NR_147303 |
| *Pseudocercospora nogalesii* | CBS 115022 | *Chamaecytisus prolifer* | New Zealand | JQ324960 | EF394858 |
| *Pseudocercospora purpurea* | CBS 114163 | *Persea americana* | Mexico | GU253804 | GU269783 |
| *Virosphaerella irregularis* | CBS 123242; CPC 15408 (T) | *Eucalyptus* sp. | Thailand | MH874810 | NR_156518 |
| *Hyalozasmidium aerohyalinosporum* | [CBS:125011 (T)](http://www.cbs.knaw.nl/collections/BioloMICS.aspx?Fields=All&ExactMatch=T&Table=CBS+strain+database&Name=CBS+125011) | *Eucalyptus tectifica* | Australia | NG_059440 | NR_156220 |
| *Xenomycosphaerella diplazii* | CPC:24691 (T) | *Diplazium* sp. | Brazil | NG_059579 | NR_154505 |
| *Rachicladosporium inconspicuum* | CCFEE 5456 (T) | Rock | Alps | NG_059443 | NR_144966 |
| *Rachicladosporium eucalypti* | [CBS:138900 (T)](http://www.cbs.knaw.nl/collections/BioloMICS.aspx?Fields=All&ExactMatch=T&Table=CBS+strain+database&Name=CBS+138900) | *Eucalyptus globulus* | Addis Ababa,Ethiopia | NG_070537 | NR_155718 |
| *Rachicladosporium corymbiae* | [CBS:145087 (T)](http://www.cbs.knaw.nl/collections/BioloMICS.aspx?Fields=All&ExactMatch=T&Table=CBS+strain+database&Name=CBS+145087) | *Corymbia citriodora* | Ghana | NG_067850 | NR_161143 |

(T) Type specimen, (ET) Extype specimen, (E-T) Epitype specimen
